# Resilience to depression: Implication for psychological vaccination

**DOI:** 10.3389/fpsyt.2023.1071859

**Published:** 2023-02-14

**Authors:** Qin Dai, Graeme D. Smith

**Affiliations:** ^1^Department of Medical Psychology, Army Medical University, Chongqing, China; ^2^School of Health Sciences, Caritas Institute of Higher Education, Hong Kong, Hong Kong SAR, China

**Keywords:** depression, resilience, psychological vaccination, real-world natural-stress vaccination, clinical vaccination

## Abstract

From the vulnerability perspective, we often ask the question “why someone suffers from depression?” Despite outstanding achievements along this line, we still face high occurrence or recurrence and unsatisfied therapeutic efficacy of depression, suggesting that solely focusing on vulnerability perspective is insufficient to prevent and cure depression. Importantly, although experiencing same adversity, most people do not suffer from depression but manifest certain resilience, which could be used to prevent and cure depression, however, the systematic review is still lack. Here, we propose the concept “resilience to depression” to emphasize resilient diathesis against depression, by asking the question “why someone is exempted from depression?” Research evidence of resilience to depression has been reviewed systematically: positive cognitive style (clear purpose in life, hopefulness, et al.), positive emotion (emotional stability, et al.), adaptive behavior (extraversion, internal self-control, et al.), strong social interaction (gratitude and love, et al.), and neural foundation (dopamine circuit, et al.). Inspired by these evidence, “psychological vaccination” could be achieved by well-known real-world natural-stress vaccination (mild, controllable, and adaptive of stress, with help from parents or leaders) or newly developed “clinical vaccination” (positive activity intervention for current depression, preventive cognitive therapy for remitted depression, et al.), both of which aim to enhance the resilient psychological diathesis against depression, through events or training. Potential neural circuit vaccination was further discussed. This review calls for directing attention to resilient diathesis against depression, which offers a new thinking “psychological vaccination” in both prevention and therapy of depression.

## 1. Background

Globally, depression is a commonly occurring mental disorder (1). Developed in seminal work, the diathesis-stress model suggests that, under stressful circumstances, individuals with depressive diathesis (e.g., latent negative or depressogenic self-schemas: negative view about self) might suffer from depression more readily (vulnerability) (2, 3). For many years, human depression has been mostly examined from the perspective of vulnerability, which has confirmed the risk factors of depression at biological, psychological, and social levels (4–15). Despite best efforts to target vulnerability in the prevention and treatment of depression for decades, the occurrence (3–22.5%) (16) and recurrence of depression (60% after 5 years, 67% after 10 years, and 85% after 15 years) (17) of depression remain high, which suggest that focusing on vulnerability perspective is insufficient in itself to prevent and cure depression. Clearly, we need better resolution.

Despite experiencing similar life experiences or adversity, only some individuals will suffer from depression, whereas others remain psychological health, manifesting a kind of resilience (18). Original research about resilience focused on physical resilience, an ability of a material to withstand stress without cracking (from the Oxford English Dictionary). Recently, psychologists have used this term to represent psychological resilience to stress (19). As depression is a stress-related emotional disorder, we raised the concept of resilience to depression here, to emphasize the resilient diathesis in prevention and therapy of clinical depression, and to further discuss the possible ways to improve it such as “psychological vaccination” in both clinical and real-world settings. In fact, paying attention to “resilient diathesis” against depression aligns with the rationale of positive psychology (20), which emphasizes the positivity of people (e.g., happiness, hopefulness). Thus, to explore depression from the resilient perspective may suggest a new way of thinking toward prevention and treatment of depression.

## 2. Resilience to depression

Based on diathesis-stress model, although some depression cases are causally caused by stressful life-events, however, these events only raise a moderate risk for depression (21), while personal diathesis are indispensable endogenous attribute factors. Here we focus on the resilient diathesis to discuss how this part of personal diathesis may act against depression, since it has been reported being important in fighting against adversity (22).

### 2.1. Basic assumptions of resilience to depression

The first basic assumption of resilience to depression hypothesis is that resilience is a kind of stable personal diathesis. Resilience is often manifested in biological, psychological, and social levels (23, 24). Among which, people are easily convinced that biological and psychological factors are belonging to personal diathesis, while social factors are usually viewed as external factors which influence resilience as exogenous environmental variables. Werner’s longitudinal data (22) indicated that resilient children were usually lucky: they have a closer bond with a supportive supervisor, parent, or other mentor-like person. However, the fact is that the resilient children display temperamental characteristics that elicited positive responses from their caregivers since infant. That is to say, the reason for lucky resilient children having supportive caregiver is mainly due to their positive social orientation and good communication skills instead of luck. Thus, the most reliable claim is that positive social orientation and good communication skills result in closer social interaction manifested in resilient children, which could be interpreted as a diathesis rather than luck.

The second assumption is that resilience to depression is a dynamic process rather than a static status. In Werner’s thirty-2-years longitudinal investigation, some children were overwhelmed by strong stresses at one time and their resilience crashed. However, they were able to recover in later life and be resilient as much as those who were resilient the whole way through (22). This means that the resilient diathesis against depression is relatively stable but dynamic and could be enhanced.

The third basic assumption is that, in general, there should be more depression-resilient individuals vs. depression-vulnerable individuals. The evidence is obvious. Disasters and stressful life-events happen often. However, most people who experience negative life-events remain psychologically healthy without falling into depressive illness, since that the occurrence of depression in whole population is around 3–22.5% (16). In other words, most people would not suffer from depression, despite of the fact that they have experienced severe negative events, i.e., resilience is an ordinary magic in general population (25).

The fourth assumption is that resilience to depression is equivalent in male and female. Although quite a few studies have reported that depression is twice as common in women than in men (26, 27). However, our previous work has proposed an alternative hypothesis “Gender differences in self-reporting symptom of depression” (28), suggesting that mild-moderate depression tends to be reported more often by females, and severe depression and suicide tend to be reported more often by males. Potential mechanisms that account for this difference have been discussed from biological, psychological and social aspects. This newly proposed hypothesis is to emphasize that male depression is under-diagnosed and under-treated, rather than that males suffer from depression less often. See this perspective for details (28).

### 2.2. Definition of resilience to depression

Based on literature exploration, previous review affirms that *psychological resilience* is “the ability to adapt positively to life conditions. It is a dynamic process evolving over time that implies a type of adaptive functioning that specifically allows us to face difficulties by recovering an initial balance or bouncing back as an opportunity for growth”(29). Resilience has a complex construct that can be conceptualized as a *trait* being possessed with varying degrees in different individuals, a *state* with bidirectional relationship with environmental and developmental variables, and as an *outcome* of confronting with stress or adversity (30).

Thus, how to define the resilience to depression? Basically, it has been assumed that depression-resilient individuals are less likely to suffer from depression even they are confronted with certain types of stress (severe life experiences or chronic everyday stresses) (31). This group of people possess several characteristics: more positive attributional style, lower anxiety, more social activities, been accompanied more by caregivers (32, 33), et al. They manifest a resilient diathesis against depression (weak depressive diathesis). Thus, combined the literature together, the *resilience to depression* could be described as: under chronic daily life difficulties or stressful life events, individuals with weak depressive diathesis show a certain level of stress responses with or without functioning impairment. However, they are exempted from typical depressive symptoms, and could not be diagnosed as either kind of depressive disorders based on the Diagnostic and Statistical Manual of Mental Disorders V (DSM-V) (34).

### 2.3. Comparison of resilience to depression with previous theories

Although depression has been identified for many decades and resilience has been emphasized recently, however, the two topics are mainly discussed separately. The “resilience to depression” presented here is distinct from previous resilience or depression theories by combining them together.

#### 2.3.1. Resilience to stress

Currently, most studies about resilience focus on resilience to stress (35, 36). Based on the definition of “psychological resilience” (29), Masten characterized resilient individuals by identifying those who experienced significant life-adversities but never showed significant psychological dysfunction or psychopathology (25). Recently, DiCorcia argued that resilience could be developed successfully from not only the severe life experiences but also the everyday stressors (23). Together, resilience to stress means good outcomes in psychology (adaptation or development) despite of serious threats or everyday stress (23, 25).

Poor resilience to stress (high vulnerability to stress) may result in several emotional disorders, such as anxiety or depression (37). Depression is a stress-induced (related) emotional disorder (38–40). It is almost impossible to discuss depression without referring to stress. Thus, a naturally raised question is the relationship between resilience to stress and resilience to depression. Obviously, resilience to stress and resilience to depression is highly correlated, individuals with high levels of resilience to stress are more likely to be exempted from depression (33, 41), whereas individuals with high vulnerability to stress have a higher possibility of suffering from depression under adversity (37). However, resilience to stress and resilience to depression are not equal. On the one hand, animal experiments showed that animals vulnerable to depression-like behavior (e.g., sucrose preference <65%) displayed a unique molecular profile when compared to *resilient to depression-like behavior but anxious* animals. This suggests that *resilience to depression* does not mean resilience to stress since that anxiety is a common kind of stress response and psychological impairment (*vulnerability to stress*) (7). On the other hand, human studies indicated that compared with persons vulnerable to stress, individuals with high resilience to stress may find it easier to recover from depression, responding well to antidepressant treatment (42, 43). This suggests that individuals with high *resilience to ordinary stress* may suffer from depression under severe adversity, manifests a *vulnerability to depression*.

In summary, resilience to stress and resilience to depression appear highly correlated, but different. Resilience to stress is a much broader term related to stress-relevant general mental health and adaptive functioning, whereas resilience to depression is a more specific term toward a kind of stress-induced emotional disorders—depressive disorder. Diathesis belonging to resilience to stress and resilience to depression may be overlapped in some parts, but been differentiated in other parts.

#### 2.3.2. Diathesis-stress model

For decades, the diathesis-stress model has been accepted widely, placing emphasis on the diathesis which is more likely developed into depression under stress (vulnerability) (44, 45), that is, “why someone suffers from depression.”

The concept of “resilience to depression” presented here pays attention to the diathesis which less likely leads to depression (resilience), that is, “why someone is exempted from depression.” Studies indicated that depression-resilient individuals were less likely to suffer from depression (33) and with lower suicidal rates (46, 47). The results suggest that some features may protect people from depression (48). Agreement on this idea has led to more thinking toward the prevention and treatment of depression. That is, depression may be prevented effectively if psychologists could fully identify the resilient trait against depression in individuals and transplant into depression-vulnerable persons (i.e., decrease the occurrence of depression). Similarly, if clinical psychiatrists could introduce resilience traits into therapy of depression, the cognitive and behavioral model of patients might be armed with the resilient model, which speeds the recovery of clinical depression.

#### 2.3.3. Protective factors of depression

Based on recent review, protective factors of depression have been explored mainly from sociodemographic factors, physical factors, biological factors, lifestyle factors, and psychological factors (49). As discussed in the first paragraph of 2.1, a basic assumption of resilience to depression hypothesis is that resilience is a kind of stable personal diathesis, which is often manifested in biological, psychological, and social levels. In which, most biological (e.g., neurotransmitter) and psychological (cognition, emotion, et al.) variables may be shared by both protective factors of depression and resilience to depression. While most sociodemographic variables (e.g., age, gender, and education), physical factors such as weight, and lifestyle factors (e.g., dietary patterns, smoking and alcohol consumption) may only belong to protective factors of depression, since they are unstable factors which influence the resilience to depression dynamically (50). And for social variables (e.g., stressful life-events), part of them may belong to protective factors of depression, and others belong to resilience to depression. For example, less stressful life-events are protective factors of depression (51), however, which is not a resilient trait against depression as generally believed. Another study indicated the role of absence of childhood history of sexual abuse in the prevention of depression (52), however, which is not a resilient trait of depression too, since it is not an inner diathesis but a hardly controlled outer environment. Moreover, social support includes three parts, i.e., objective support, subjective support, and utilization of the support (53). In which, subjective support (e.g., How many closer friends you have, from whom you can obtain support and help) and utilization of the support (e.g., Who are you turning to help when you are in trouble) may belong to both resilience to depression and protective factors of depression, while objective support (e.g., In the past, when you were in crisis, your obtained financial or problem-solving support included) may be only a part of protective factors of depression but not a resilient trait of depression.

In sum, protective factors of depression include inner and outer factors protecting people from depression which influence the level of resilience to depression together with the risk factors of depression dynamically, while resilience to depression focuses on inner stable personal trait against the occurrence and recurrence of depression.

## 3. Research evidence for resilience to depression

**FIGURE 1:**
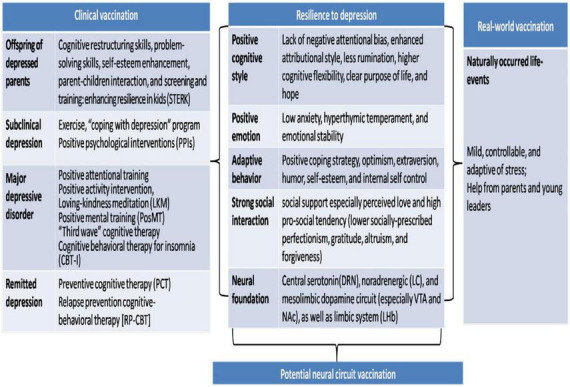
Research evidence for resilience to depression and implication for psychological vaccination. Research evidence for resilience to depression has been reviewed systematically: positive cognitive style, positive emotion, adaptive behavior, strong social interaction, and neural foundation. Inspired by these evidence, “psychological vaccination” could be achieved by real-world natural-stress vaccination or newly developed “clinical vaccination,” both of which focus on enhancing the resilient psychological diathesis against depression, through learning or life-events. Potential neural circuit vaccination has been discussed further.

How to recognize the phenotype of resilience to depression? The evidence will be reviewed^1^ systematically from the following five perspectives (Figure 1): positive cognitive style, positive emotion, adaptive behavior, strong social interaction, and neural foundation.

### 3.1. Positive cognitive style

At the cognition level, *the styles in cognitive process* (e.g., attention, interpretation, attribution, or memory), *cognitive regulation*, *cognitive flexibility*, and *cognitive attitude* (e.g., purpose of life or hopefulness) have been indicated to be involved in resilience. Specifically, there was a negative correlation between trait resilience and *attentional bias* to threat when the attentional control was low, while there was a positive association between trait resilience and attentional bias to threat when the attentional control was high (54), which suggested a dynamic relationship between trait resilience and negative attentional bias. Studies further found that individuals with an enhanced *attributional style* reported higher positive affect (55). They were more resilient to the development of depressive symptoms conferred by negative events (56) or hopelessness (57). Indeed, the strongest support of resilience was found for more positive attributional style (58). In addition, the *cognitive regulation strategies* of refocusing on planning and less rumination contributing to resilience in patients with depression have been reported (59). In relation to *cognitive flexibility*, people with poorer cognitive flexibility may not find cognitive restructuring as useful to alleviate depression as those with better cognitive flexibility (60). Moreover, positive *cognitive attitude* has been indicated a promoter of resilience. For example, higher levels of purpose in life predicted resilience in U.S. military veterans, which suggested that a clear purpose in life helps to fight against depression (61). Similarly, hopefulness turned out to be the strongest negative correlates of anxious/depressive symptoms (62). The results suggest that a lack of negative attentional bias, enhanced attributional style, less rumination, higher cognitive flexibility, clear purpose of life, and hopefulness are correlated with resilience to depression.

### 3.2. Positive emotion

At the emotional level, *less negative emotion* (e.g., *anxiety*), *stronger positive emotion*, and *higher emotional stability* have been indicated contributing to resilience. Specifically, lower levels of *anxiety* or perception of stress, might protect against chronic insomnia in people vulnerable to stress-related insomnia (63). Conversely, a high anxiety personality trait has been recognized as a vulnerability factor to the development of depression (10). Indeed, anxiety has been constantly present in low resilience. Animal-model evidence further confirmed that high anxiety led to vulnerability to stress-related depression-like behavior, whereas low anxiety led to resilience (32). Moreover, it was indicated that there was a strong relationship between *hyperthymic temperament* and psychological resilience in individuals with major depressive disorder (43). Isaacs’s representative investigation of 2157 U.S. military veterans showed that higher levels of *emotional stability* predicted resilience (61). These results support the role of positive emotion (64) (especially low anxiety, hyperthymic temperament) and emotional stability in resilience to depression.

### 3.3. Adaptive behavior

At the behavioral level, coping strategy and personality (e.g., optimism, extraversion, humor) including self-related personality trait (e.g., self-esteem, self-control) are most important (65, 66). For *coping strategy*, problem-solving/approach coping strategies are correlated with a decreased risk for depression (resilience), while emotion-focused/avoidant coping strategies are correlated with an increased risk for depression (67). Furthermore, self-blame were positively correlated with depression, whereas positive refocusing, positive reappraisal, acceptance, and planning were negatively correlated with depression (65). For *personality trait*, it was confirmed that persons with higher optimism have lower risk (resilience) for suicidal ideation and suicidal attempts in front of low to moderate negative life-events (66). Similarly, higher levels of extraversion predicted resilient status in Isaacs’s investigation of U.S. military veterans (61). In addition, humor-based strategies had adaptive consequences in remitted depression (68). Referring to *self-related personality trait*, latest review concluded the strongest support of resilience as higher self-esteem (58). Werner’s seminal longitudinal investigation (69) indicated the importance of an internal locus of control in resilient children: they believed that themselves instead of their citations decided their achievements. The resilient children saw themselves as the orchestrators of their own life. They tended to “meet the world on their own terms.” These findings confirm the involvement of positive coping strategy, optimism, extraversion, humor, self-esteem, and internal self-control in the resilience to depression.

### 3.4. Strong social interaction

At the social level, social support and pro-social tendency (e.g., gratitude, altruism, forgiveness) have been indicated promoting the resilience to depression. *Social support* represents perceived affection, empathy, love, concern, trust, intimacy, acceptance, encouragement, or caring from others (70), which is important to maintain personal mental health. Indeed, depression-resilient individuals showed more social activities, and were accompanied more by caregivers (33). Particularly, the older individuals without major depression had better family function and higher social support than olders with depression (71). Moreover, studies from undeveloped countries indicated the effect of enhanced social support on reducing community-based depressive symptoms (72). This effect was most significant in women (73), including those who suffered from family violence (74, 75). In addition, one international survey indicated that perceived love and interconnectedness were associated with less risk of psychopathology in China, India, and the United States (76). Indeed, based on the 76 years longitudinal Grant Study, the warmth of childhood relationship with parents has the greatest positive impact on “’life satisfaction” (77). Referring to *pro-social tendency*, recent review concluded that the strongest support of resilience was generated from lower socially prescribed perfectionism (58). Importantly, higher levels of dispositional gratitude (61) and altruism (76) were constantly reported correlating with higher resilience and lower risk of psychopathology. Similarly, forgiveness turned out to be the strongest negative correlates of anxious/depressive symptoms (62). Thus, social support especially perceived love and high pro-social tendency (lower socially prescribed perfectionism, gratitude, altruism, and forgiveness) have been identified as resilient factors of depression (13, 14).

### 3.5. Neural foundation of resilience to depression

Why someone shows resilience after adversity while others do not? Neural alteration might underlie the behavioral phenotype of resilience. Latest review indicates a correlation between personality traits and emotions, and both depend on monoamine neurotransmitters (dopamine, norepinephrine and serotonin) (78). Fundamental and clinical studies have demonstrated that decreased serotonin (5-HT) (79) and noradrenergic (NE) (80) are closely correlated with depression. Specifically, animal studies showed that central 5-HT was important in resilience toward adversity (7, 81). Additionally, variations in the serotonin transporter gene (5-HTTLPR) have also been identified as associated with resilience to depression through their effects on social cognition (82). Dorsal raphe neurons (DRN) played an important role in serotonin transmission to amygdale, hippocampus, lateral prefrontal cortex (PFC), nucleus accumbens, and hypothalamus (83), and deletion p38α MAPK in DRN induced resilience phenotype in mouse (84). Moreover, NE neurons in the locus coeruleus (LC) regulated resilience to social defeat through inhibitory control of dopamine neurons in ventral tegmental area (VTA) (80). Indeed, the mesolimbic dopamine (DA) circuit was identified in resilience recently, which was correlated with sucrose preference behavior (85), and uniquely associated with vulnerability or resilience to depression-related behavior. Specifically, it was indicated that VTA and the nucleus accumbens (NAc) area of the brain were correlated with resilience (86). About the cerebral circuit, *in vivo* evidence with optogenetic methods suggested the differences in projection-pathway specificity in promoting resilience, optogenetic inhibition of the VTA—medial PFC projection promoted vulnerability, whereas inhibition of the VTA–NAc projection induced resilience (85). Interestingly, the activation of midbrain, dorsal and ventral striatum, medial prefrontal, and right orbitofrontal cortices were also strongly correlating with human positive emotion—love (87). Study also found that βCaMII expression was significantly up-regulated in the lateral habenula (LHb) of animal models of depression, and down-regulated by antidepressants (88), which identify βCaMKII as a key molecular determinant of vulnerability or resilience to depression. The evidence supports the view that central serotonin (DRN), noradrenergic (LC), and mesolimbic dopamine circuit (especially VTA and NAc), as well as limbic system (LHb) are cerebral basis underling resilience to depression.

## 4. Psychological vaccination: Implications to prevent and cure depression

Is that possible to transform a person from vulnerable to depression into resilient against depression? Masten has said that resilience is ordinary magic and that it usually arises from the normative functions of human adaptation system (25). This provides a positive perception on improvement of the resilient diathesis in human beings: it is possible and feasible to improve resilience against depression through “psychological vaccination” in both clinical and real-world setting. “Psychological vaccination” is similar with biological vaccination, such as the influenza vaccination, during which, resilient diathesis is enhanced through learning or stressful life-event (Figure 1).

### 4.1. Real-world natural-stress vaccination

#### 4.1.1. Negative events make people psychologically stronger

Friedrich Wilhelm Nietzsche once said: “That which does not kill us makes us stronger.” The involvement of environment, especially the early childhood experiences, in the development of psychological resilience, have been well established (22). According to the empirical evidence, it has been argued that childhood stress exposure did not increase the vulnerability to stress-related psychopathology (e.g., depression) as a linear function, but instead reflected as a quadratic function (89). Indeed, unsuccessful adaptations of childhood life-events especially childhood abuse and trauma might result in psychological trauma such as depression (48). However, stressful events that were effectively adopted in short-term and reiterated over the long-term increased children’s as well as adults’ capacity to cope with more intense stressors (23). Similarly, it was confirmed that individuals with more aversive childhood events reported higher compassion satisfaction and lower rates of burnout in adulthood (90). Together, the effect of early life-events on resilient diathesis is rather result-dependent: maladaptation results in function impairment or psychological trauma and blocking the development of resilience, whereas successful coping leads to mastery in the face of stress or adversities without function impairment or trauma and enhancing the resilient diathesis. During which, adequate coping style (67) or optimism personality (66) or social support (33) may act as intermediaries. That is “every cloud has a silver lining” or “something good can come from misfortune.”

#### 4.1.2. Way for real-world natural-stress vaccination

Real-world natural-stress vaccination means that exposure in mild stress causes no depression, but makes people more resilient to more stressful situation (been stronger psychologically), i.e., the resilient diathesis to depression is enhanced naturally. This process is similar with the biological vaccination, such as the influenza vaccination, supporting the view that “one cannot become a master sailor on calm seas.” Based on the quotidian resilience model, in the real-world life, mild, controllable, and adaptive of naturally occurred events are critical in obtaining the resilient diathesis (23), since that the adversity in the real-world is unavoidable. Particularly, there are different types of stress (81), individuals may be able to cope very well with some types of stresses or difficulties, but deal much poorly with others. Thus, for different stresses, different levels of efforts are needed for different people. Moreover, children’s resilience could be enhanced *via* a good mentor-student relationship (91). Significantly, it was reported that infants made more efforts to reach a goal when they saw adults persist and that infants could generalize the value of persistence to new tasks (92). Thus, it is necessary to involve parents or young leaders in real-world natural-stress vaccination when life-events occurred naturally during children’s growing up (93). As a result, the resilient diathesis of young children may be enhanced more effectively.

Moreover, animal experiment confirmed that *artificial given early moderate stress* strengthened socioemotional and neuroendocrine resistance to subsequent stressors in monkeys (94). Animal models of stress inoculation may offer new views for preventive or therapeutic strategies of human stress-related psychiatric disorders such as depression (95). However, mainly due to the ethic issue, artificial given stress (stress inoculation) were only practiced in animals. Hopefully but carefully, after the causal relationship between stress and resilience is fully revealed in animals, the involvement of artificially given stress in stress vaccination in people could be expected. Like biological vaccination, the stress applying for “psychological vaccination” in people should be tested strictly in controlled experimental animal first and then conducted on a real-world person. It is also important to stress that any such study have to fully comply with the ethical principles.

### 4.2. Clinical vaccination

#### 4.2.1. Resilience could be trained

Besides real-world natural-stress vaccination, recent studies have suggested that individuals can learn “resilience” through mentoring or coaching without stress exposure (96), which could be a much safer and easier way to foster resilient diathesis. Indeed, Werner’s seminal study reveals that children who were not resilient in their early years became more resilient when they knew more about the skill of resilience (22). This clearly suggests that resilience could be trained through practice.

#### 4.2.2. Ways for clinical vaccination

The basic idea of “clinical vaccination” is that: the resilience traits (*positive cognitive style*, *positive emotion*, *adaptive behavior*, and *strong social interaction*) can be incorporated into resilience training programs, and subjected to depression-vulnerable persons or depressed patients, which could help to enhance resilient diathesis, and acts against the occurrence or recurrence of depression.

##### 4.2.2.1. High risk offspring with depressed parents

In a 30-years longitudinal investigation, it was found that biological offspring with two previous generations who suffered from major depression were at higher risk for major depression (97). For the high-risk population, the intervention emphasis was put on the training of long-term cognitive-behavioral model. Specifically, intervention project for the offspring of depressed parents focused on cognitive restructuring skills toward negative thoughts, and problem-solving skills for stress (98). Recent review highlighted the importance of parent-children interaction in this population (99), which were incorporated into the project Screening and Training: Enhancing Resilience in Kids (STERK) (100). The results suggested that the cognitive restructuring skills, problem-solving skills, and parent-children interaction, are effective targets in the resilience interventions of offspring with depressed parents.

##### 4.2.2.2. Subclinical depression

Subclinical depression is a normal emotional status been experienced by almost all people during life span, which might be developed into clinical depression if failed in adjustment (101). Earlier study suggested that exercise training generated long-lasting resilience to stress in subclinical depression (102). Based on this, researchers explored and raised “Coping with Depression” program to further improve the resilience in individuals with subclinical depressive status (103). Furthermore, positive psychological interventions (PPIs) and wellbeing–enhancing activities were also indicated effectiveness in subclinical depression (104, 105). The results highlighted the prevention strategies (exercise, “Coping with Depression” course, and PPIs) in enhancing the resilience of subclinical depression.

##### 4.2.2.3. Major depressive disorder (MDD)

MDD is a mood disorder which has been classified in DSM-5 (106). For clinical depression, more systematic and multidimensional intervention skills were designed to alleviate symptoms and boost resilience. Horner and colleagues found that both depressed and non-depressed individuals responded positively to happy scripts, but depressed individuals cannot achieve or sustain equivalent levels of happy affect, which suggested that enhancing resilience to depression might focus on increasing the ability to engage in positive stimuli over a sustained period (107). Indeed, positive activity intervention (PAI) has been designed to improve the resilience to depression by targeting the positive affect system (108). Besides, a positive attentional training program (toward positive and away from negative stimuli) is practicing with a long-term (1 year) effect in adult outpatients with MDD (109). In total, Positive Mental Training (PosMT) represented good value in treatment of clinical depression (110).

Moreover, it was found that “third wave” cognitive therapy including dialectical behavior therapy, schema therapy, acceptance and commitment therapy, meta-cognitive therapy, and mindfulness—based cognitive therapy, might be effective ways to alleviate depressive symptoms compared with mentalization-based treatment (111, 112). Indeed, loving-kindness meditation (LKM) has a large effect on self-reported and clinician-reported reduction in depression (113). Furthermore, it was found that general sleep quality was significantly improved in patients who received cognitive behavioral therapy for insomnia (CBT-I), which suggests that CBT-I might be an useful strategy to improve sleep quality in patients with depression and comorbid insomnia (114). The results suggest the effectiveness of focusing on reducing negative emotion and building positive emotion in boosting resilience in patients with MDD (115).

##### 4.2.2.4. Remitted depression

First occurrence of depression are more likely than recurrences to follow severe life-events (116, 117), which suggested that the increased vulnerability or decreased resilience might play an important role in the recurrence of depression. Thus, the recurrence rate of depression might be reduced effectively by enhancement in resilient diathesis (64). Indeed, it was found that the Preventive Cognitive Therapy (PCT) in remitted patients with multiple prior episodes had long-term (10 years) preventive effects on recurrence (118). Very similar, an additional relapse prevention cognitive-behavioral therapy [RP-CBT] after acute response to antidepressant had a continued effect on reducing the risk of relapse (119). Thus, to enhance resilience in patients remitted from depression, recurrent preventing and enhancing positive memories are critical.

## 5. Open questions and future directions: Potential neural circuit vaccination

Besides well-known natural-stress vaccination and “clinical vaccination,” can we go further with the knowledge of resilience to depression? Potential neural circuit vaccination should be under consideration.

### 5.1. Brain function targeted cognitive training

It was indicated that compassion training increased positive affective experiences (resilience phenotype), elicited activity in a reward network associated with positive affect and affiliation (120). The findings were replicated later (121), which suggested that reward network could be enhanced along with the improvement in emotional resilience. The results suggest a possibility of neural network vaccination through cognitive training.

### 5.2. Physical stimulation

Recently, fruitful advances in neuroscience lead to huge zest in exploration of invasive, precise, and effective neurobiological intervening methods to cure psychological disorder. Among which, transcranial direct current stimulation (tDCS), has been considered as effective means to buffer cognitive functions or neural plasticity, the concurrent applications of tDCS during cognitive training may potentially facilitate short- and long-term cognitive and brain plasticity (122). Moreover, multiple-day targeted repeated transcranial magnetic stimulation (rTMS) of hippocampal-cortical networks produced a long-lasting enhancement in the ability to learn novel face-word pairings, and increased functional connectivity of the targeted portion of the hippocampus with distributed regions of the posterior hippocampal-cortical network (123). Recent studies also confirmed the positive effects of rTMS on stress resilience which underscore the possible benefit of high frequent-rTMS as a transdiagnostic intervention (124). The results highlighted a possibility of neural circuit vaccination through magnetic or current stimulation in clinic, which needs more evidence and has a long way to go.

In conclusion, we propose the concept “resilience to depression” here, to better emphasize the diathesis that is less likely developing depression, that is, “why someone is exempted from depression.” The concept presented here guides the attention of psychologist and psychiatrist to the resilient diathesis against depression, which suggests a new thinking “psychological vaccination” in prevention as well as therapy of depression. If people could successfully adapt the naturally occurred mild controllable events (with the support of mentor), i.e., *real-world natural-stress vaccination*, the occurrence of depression might be reduced and better prevented. Alternatively, if individuals at high risk for depression or in clinical depression could make effective cognitive or behavioral modification through resilience training program, i.e., *clinical vaccination*, the individuals at high risk for depression will be exempted from depression in their later lives, or the patients will be cured more completely with a lower recurrent rate.

## Author contributions

QD drafted the manuscript. GS revised the manuscript. Both authors contributed to the article and approved the submitted version.

## Footnotes

1 To ensure that took as systematic approach to the literature review, we searched six databases (PubMed; Medline; EMBASE; Cochrane; EBSCO Psyc ARTICLES; Web of science) and three of the largest databases in China (CNKI; Sinomed; Weipu; WANFAN DATA) to find articles related to this topic. The main key words for searching were as follows: resilience/resilient AND depression (depression or depressive or major depressive disorder) AND/OR one of the five perspectives [COGNITION: cognitive style or cognitive process attentional bias or attributional style or interpretation bias or memory bias or cognitive regulation or cognitive flexibility or cognitive attitude; EMOTION: emotion or anxiety or emotional stability; BEHAVIOR: behavior or coping or personality trait; SOCIAL INTERACTION: social support or social interaction or interpersonal interaction or interconnectedness or pro-social tendency; NEURAL FOUNDATION: neural transmitter or serotonin (5-HT) or noradrenergic (NE) or dopamine (DA) or cortex or circuit or ventral tegmental area (VTA) or nucleus accumbens (NAc) or Dorsal raphe neurons (DRN) or prefrontal cortex (PFC) or locus coeruleus (LC) or lateral habenula (LHb)]. For example, one of the searching strategies was: (“resilience” AND “depression” AND “attentional bias”).
